# Laminin enhances the growth of human neural stem cells in defined culture media

**DOI:** 10.1186/1471-2202-9-71

**Published:** 2008-07-23

**Authors:** Peter E Hall, Justin D Lathia, Maeve A Caldwell, Charles ffrench-Constant

**Affiliations:** 1 Department of Pathology, University of Cambridge, Cambridge, UK; 2 Cambridge Centre for Brain Repair, University of Cambridge, Cambridge, UK; 3 Laboratory of Neurosciences, National Institute on Aging, Baltimore, USA; 4 Laboratory for Integrative Neuroscience and Endocrinology, University of Bristol, Bristol, UK; 5 MRC Centre for Regenerative Medicine, Queen's Medical Research Institute, Edinburgh, UK

## Abstract

**Background:**

Human neural stem cells (hNSC) have the potential to provide novel cell-based therapies for neurodegenerative conditions such as multiple sclerosis and Parkinson's disease. In order to realise this goal, protocols need to be developed that allow for large quantities of hNSC to be cultured efficiently. As such, it is important to identify factors which enhance the growth of hNSC. *In vivo*, stem cells reside in distinct microenvironments or niches that are responsible for the maintenance of stem cell populations. A common feature of niches is the presence of the extracellular matrix molecule, laminin. Therefore, this study investigated the effect of exogenous laminin on hNSC growth.

**Results:**

To measure hNSC growth, we established culture conditions using B27-supplemented medium that enable neurospheres to grow from human neural cells plated at clonal densities. Limiting dilution assays confirmed that neurospheres were derived from single cells at these densities. Laminin was found to increase hNSC numbers as measured by this neurosphere formation. The effect of laminin was to augment the proliferation/survival of the hNSC, rather than promoting the undifferentiated state. In agreement, apoptosis was reduced in dissociated neurospheres by laminin in an integrin β1-dependent manner.

**Conclusion:**

The addition of laminin to the culture medium enhances the growth of hNSC, and may therefore aid their large-scale production.

## Background

Neural stem cells (NSC) have the ability to give rise to the three main cell types of the central nervous system (CNS), as well as being able to self-renew. As such, both public and scientific attention has focussed on them as potential therapies for neurodegenerative diseases, including multiple sclerosis and Parkinson's disease. The scarcity of primary human tissue from which these NSC can be isolated has generated a need to develop protocols that allow their expansion in cell culture. As a first step towards this goal it is necessary to identify and understand the factors that regulate NSC growth and differentiation. Important clues as to the identity of these factors come from the observation that the NSC are not randomly distributed throughout the brain. Instead, they reside within distinct microenvironments or niches such as the ventricular/subventricular zone (VZ/SVZ) of the developing CNS and the subependymal zone (SEZ) of the adult CNS [1,2]. Studies of such niches from different tissues and organisms have led to the identification of common signalling molecules including growth factors, cell-cell signalling molecules, adhesion molecules and extracellular matrix (ECM) molecules, and combinations of these are therefore likely to provide strategies to maintain stem cell populations and so enhance NSC growth in cell culture [3,4].

The demonstration that laminins make direct contact with adult NSC via basal lamina-like extension from blood vessels in the SVZ called fractones [5,6], and with embryonic NSC via laminins expressed within the ventricular zone [7-9], highlight the likely key role of these ECM molecules in NSC regulation. Acting mainly through the integrin family of receptors, the ECM is a key regulator of all aspects of cell behaviour, including proliferation, survival, migration and differentiation [10]. Integrins are transmembrane αβ heterodimers that undergo a conformational change upon ligand binding, thereby leading to downstream effects such as actin polymerisation and synergism with growth factor signalling [10]. In other stem cell systems, the ECM has been found to inhibit the terminal differentiation of epidermal stem cells [11]. Similarly, the properties of both embryonic and spermatogonial stem cells can be maintained *in vitro *by growth on a laminin substrate [12,13]. In agreement, the elevated expression of integrins, particularly the laminin-binding α6β1 heterodimer, has been used to isolate stem cells from other, more differentiated cell types [14-20], and integrin β1 has also been implicated in the maintenance of epidermal and prostate stem cells [21,22]. In contrast, less is known about the role of laminin/integrin interactions on human NSC (hNSC). They have been shown to promote the migration of hNSC in an integrin α6-dependent manner [23]; likewise a cholinergic neuronal fate is promoted by laminin [24]. However, the role of laminins in hNSC growth has not been examined. Here, we test the hypothesis that recreating features of the niche microenvironment by the addition of exogenous laminins promotes human NSC growth and provides a strategy for improving the efficiency of their propagation for translational research use.

## Methods

### Reagents and antibodies

N2 and B27 culture supplements were purchased from Gibco (Paisley, UK). Epidermal growth factor (EGF) and fibroblast growth factor-2 (FGF-2) were obtained from R&D Systems (Abingdon, UK). Accutase was purchased from Millipore (Chandlers Ford, UK). The integrin β1-stimulatory antibody, TS2/16, was purified from the hybridoma culture supernatant (LGC-Promochem, Teddington, UK) by incubation with Protein G beads (GE Healthcare, Little Chalfont, UK) overnight at 4°C, before being eluted according to the manufacturer's instructions. Unless already sodium azide-free, antibodies used in functional studies were dialysed overnight at 4°C in phosphate-buffered saline (PBS) using sterile Slide-a-lyzer cassettes (Pierce, Cramlington, UK). The sterile mouse IgG_1 _isotype control (clone W3/25) was purchased from Serotec (Oxford, UK). Human placental laminin (hereafter referred to as "laminin") was purchased from Sigma-Aldrich, as were all other reagents (Poole, UK).

### Human cell culture

Human fetal tissue (8–10 weeks postconception) was collected in accordance with the arrangements for informed consent recommended by the Polkinghorne Committee (1989) and the UK Department of Health guidelines (1995), and with the approval of the Addenbrooke's Hospital Medical Research Ethics Committee (Cambridge, UK). The hNSC from the cortex were propagated as free-floating aggregates (neurospheres) and cellular expansion prior to use in the studies described here was achieved using a passaging method described in detail elsewhere [25]. In brief, fresh tissue was initially dissociated in trypsin and seeded at a density of 200,000 cells/ml in a T75 flask containing Dulbecco's Modified Eagle's Medium (DMEM)/HAMS F12 (3:1), supplemented with N2 (1:100), EGF, FGF-2 (both at 20 ng/ml) and heparin (5 μg/ml). Cultures were fed every 4–5 days by replacing half the medium and spheres were passaged after 14 days expansion by cutting into 200 μm sections using a McIlwain tissue chopper.

### Neurosphere assay

Human neurospheres were dissociated with accutase before live cells were sorted using a MoFlo flow cytometer (DakoCytomation, Ely, UK) into 96-well plates (Nunc, Roskilde, Denmark) at a density of 500 per well (1,500 cells per cm^2^). In the experiments using a limiting dilution assay to determine the frequency of neurosphere-forming cells, progressively decreasing numbers of cells were plated per well. Neurosphere medium (200 μl), containing EGF, FGF-2 (both at 20 ng/ml), heparin (5 μg/ml) and 2% B27 together with laminin or antibodies at the concentrations indicated in the figure legends, was added per well. Prior experiments examining the effects of different laminin concentrations on mouse neurosphere numbers showed no significant increase above 10 μg/ml (see Additional file 1 and Additional file 2), so this concentration was used throughout for the studies on human cells. Cells were fed every 4–5 days by replacing half of the medium, and the number of neurospheres formed was counted after 21 days in culture. For the purpose of this paper, these are termed primary neurospheres.

Secondary neurospheres were grown by collecting together primary neurospheres from a particular treatment group, dissociating them with accutase before re-plating at the same density using the flow cytometer in fresh medium in the absence of any antibodies or laminin. All other details are as for primary spheres.

### Conditioned medium

Conditioned medium had been exposed for 5 days to neurospheres growing in a T75 flask (see 'human cell culture'), before being passed through a 0.45 μm filter to remove cell debris. The percentage refers to the amount of conditioned medium added; fresh DMEM/Hams F12 constituted the remaining volume. An appropriate quantity of growth factors (20 ng/ml EGF and FGF-2, 5 μg/ml heparin) and supplements (1% N2 or 2% B27, as indicated in the figure legends) were added according the final volume.

### Apoptosis analysis

Human neurospheres were dissociated using accutase and grown for 24 hours in medium ± laminin (10 μg/ml). In order to examine apoptosis, cells were incubated with fluoroscein isothiocyanate (FITC) conjugated annexin V for 15 min at room temperature in binding buffer (Annexin V-FITC apoptosis detection kit I, BD Pharmingen), before being analysed on at FACScan II flow cytometer (Becton Dickinson, Oxford, UK), with at least 10000 events being collected per data file. Further analysis was performed using Summit 3.0 software (DakoCytomation).

### Statistics

For all neurosphere assays, 8 experimental replicates were performed for each tissue sample. The *n *value recorded in the figure legends refers to the number of biological replicates. The data are presented as mean ± standard error. All statistics were calculated according to the test described in the figure legends, with *p *< 0.05 considered significant.

## Results

### Human NSC will grow at clonal density in B27-supplemented medium

To test our hypothesis as to the role of exogenous laminins, we needed to develop an assay that enables single dissociated cells to reveal any stem cell properties in culture. Neurospheres provide a very widely used method to grow NSC, with each neurosphere representing a 3D aggregate generated from a single NSC and containing a mixture of NSC and more differentiated cells [26]. Human NSC are commonly grown in N2 medium containing 20 ng/ml FGF2 and EGF, and are passaged by chopping the spheres into segments to be placed in fresh medium. However this approach in which large fragments are passaged does not allow accurate quantification of the effect of laminins or other manipulations on single NSC. First, therefore, we dissociated spheres passaged by chopping into single cell suspensions and re-plated them in the same medium at 1 cell/well. No neurospheres formed in these cultures (not shown). Moreover, even when higher cell plating densities of 2000, 1000 or 500 per well (10, 5 or 2.5 per μl) were examined, extremely low neurosphere yields were observed, with only 0.036 ± 0.009%, 0.029 ± 0.007% or 0.063 ± 0.050% of the plated cells forming neurospheres, respectively (not shown).

**Figure 1:**
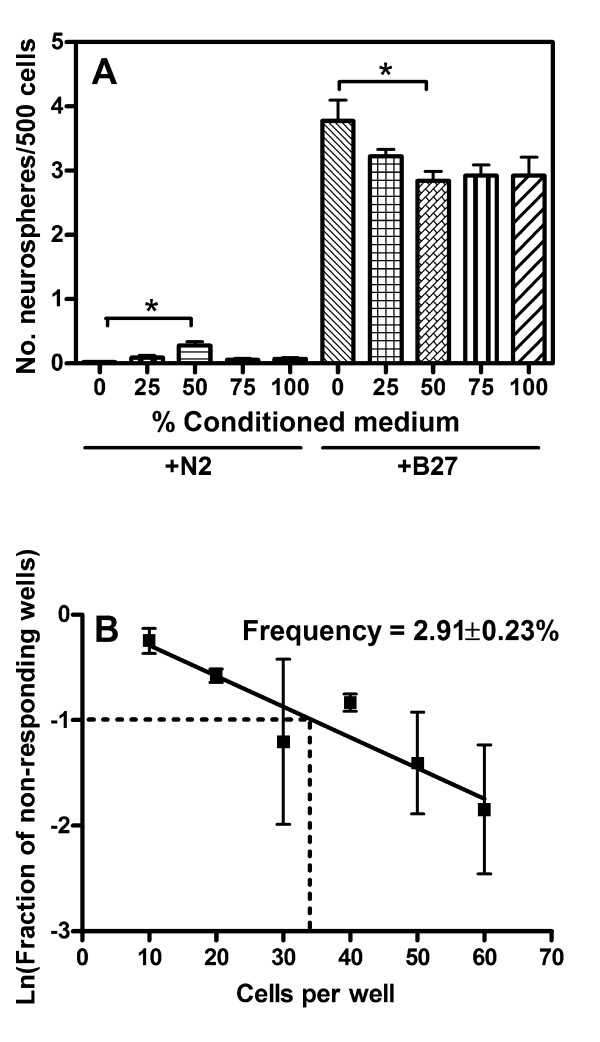
**Conditions affecting the human neurosphere assay**. (A) Dissociated neurosphere cells were plated at 500 cells per well in either 1% N2 or 2% B27 supplement, together with 0–100% neurosphere-conditioned medium. Relative to no conditioned medium (0%), a significant increase in neurosphere formation was observed for 50% conditioned medium only in the presence of N2 supplement. In comparison, 50% conditioned medium significantly reduced neurosphere formation in the presence of B27 supplement. Notably, human neurosphere formation was significantly increased by 2% B27 supplement compared to 1% N2 supplement for all concentrations of conditioned medium, as determined by a one-way ANOVA with Tukey post-test (*p *< 0.001, *n *= 2). (B) Graph showing the result of the limiting dilution assay, with cells from dissociated human neurospheres being plated at 10–60 cells per well in 2% B27 supplement. The number of non-responding wells (*i.e*. without a neurosphere present) was quantified as a fraction of the total number of wells 21 days later. Linear regression was used to produce to the line of best fit. Unless otherwise noted, statistics were determined by a one-way ANOVA with Tukey post-test. *n *= 2 for all except for the limiting dilution assay, where *n *= 3. *p < 0.05.

To ask whether these dissociated human NSC had the potential to form larger numbers of spheres given more appropriate growth conditions, we attempted to mimic the much higher cell densities present in chopped spheres by the addition of conditioned medium to the culture. 50% conditioned medium added to N2/FGF2/EGF led to a 22-fold increase in the number of primary neurospheres which formed when cells were plated at 500/well (Fig. 1A; *p *< 0.001, *n *= 2). None of the other concentrations of conditioned medium caused any significant change in neurosphere generation.

We conclude from this that the potential of the dissociated human cells to behave as NSC is much greater than as revealed by N2 medium, and that N2 does not therefore represent an appropriate choice of medium supplement for assays of human NSC behaviour and laminin effects. B27 supplement has been shown to increase the survival in culture of a range of CNS cell types, including rodent neural precursors, and is necessary to establish human neurosphere cultures from primary tissue, whereas N2 supplement is sufficient to maintain them [25,27]. Therefore, we next examined the effect of B27 supplement on the human neurosphere assay (Fig. 1A). When compared with N2 supplement, B27 led to a large and significant increase in primary neurosphere formation, with 3.775 ± 0.320% versus 0.013 ± 0.013% of cells forming primary neurospheres, respectively (*p *< 0.0001, *n *= 2). Critically, neurosphere-conditioned medium did not increase neurosphere formation in the presence of B27, with 50% conditioned medium actually leading to a decrease in neurosphere numbers (2.838 ± 0.149% versus 3.775 ± 0.320%, respectively. *p *< 0.05, *n *= 2). Consequently, these results show that B27-supplemented medium does enable human NSC to show stem cell properties in dissociated culture, and this was therefore used in all subsequent assays.

### Limiting dilution assays confirm that neurospheres provide a quantitative assay for human NSC

Prior to using neurospheres as a quantitative assay of stem cell properties, it was necessary to show that the numbers of spheres formed reflected the growth of single cells and not aggregation of two or more cells after plating. To do this, we used a limiting dilution assay (LDA) as an alternative method to the neurosphere assay for determining the frequency of the neurosphere-initiating cell in a population. By measuring the likelihood of sphere formation in progressively greater dilutions of dissociated cells, the LDA calculates the number of individual cells able to form spheres. The neurosphere-initiating frequency determined by the LDA was 2.91 ± 0.23% (Fig. 1B), which was not significantly different from the frequency calculated by the neurosphere assay (Fig. 1A; 3.78 ± 0.32%; p = 0.19, n = 3 and n = 2 for the LDA and the neurosphere assay, respectively). Therefore, as the two assays gave equivalent results, neurosphere assays were used in future experiments.

### Laminin promotes hNSC growth

**Figure 2:**
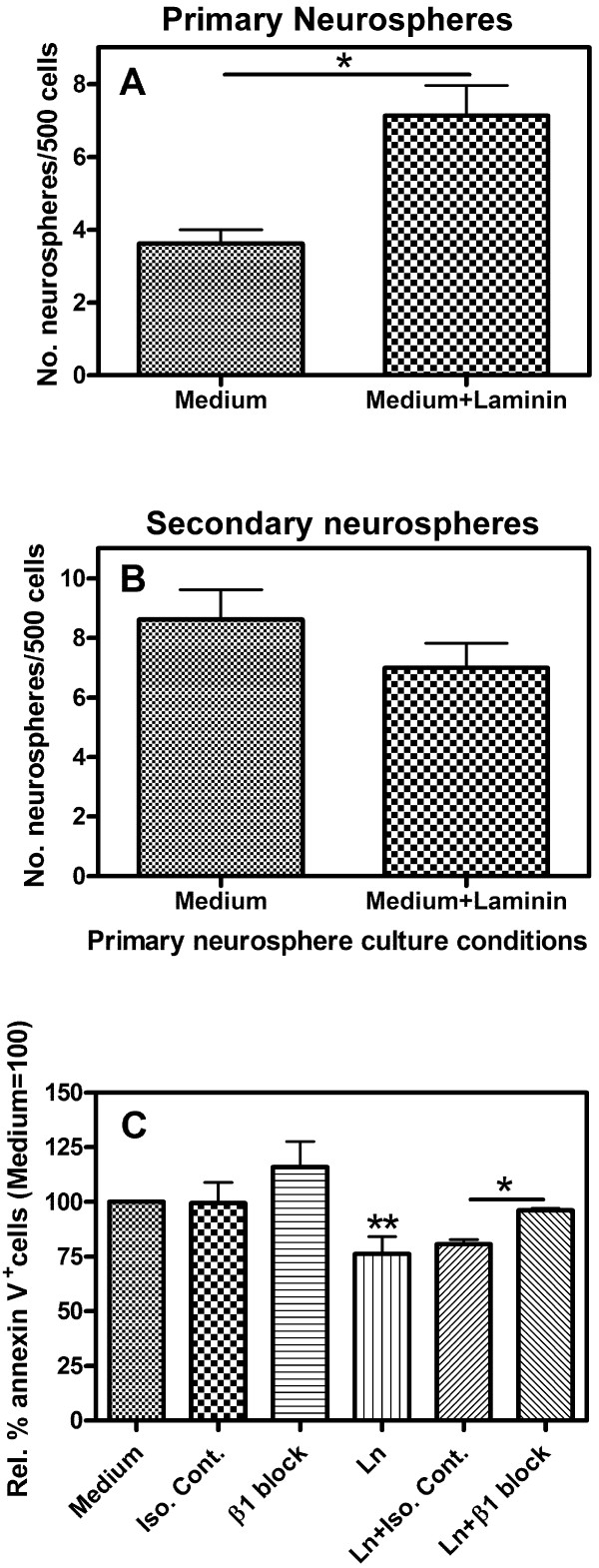
**Laminin increases human primary neurosphere formation**. (A) Cells dissociated from human neurospheres and plated in medium alone or medium containing 10 μg/ml laminin (Medium+Laminin). After 21 days, significantly more primary neurospheres had formed in the presence of laminin. (B) The neurospheres from (A) were dissociated and re-plated in medium alone. Note that no laminin was added to the culture medium in (B). No difference in secondary neurosphere numbers was observed. *p < 0.05, as determined by Student's *t*-test; *n *= 2. (C) Dissociated neurosphere cells were grown for 24 h in the absence (medium) or presence (Ln) of laminin, in addition to either an isotype control (Iso. Cont.) or integrin β1-blocking antibody (β1 block), before being analysed for annexin-V expression by flow cytometry. The number of annexin-V^+ ^cells, relative to medium alone, is shown. Annexin V-binding was significantly reduced in the presence of laminin compared to medium alone, indicating an increase in cell survival. The addition of an integrin β1-blocking antibody returned the number of annexin V^+ ^cells to control levels, indicating that laminin acts through integrin β1. Statistics were determined by a one-way ANOVA with Tukey post-test. *n *= 5 for medium and laminin, *n *= 3 for all other conditions. **p < 0.01, *p < 0.05.

Having established the conditions for the human neurosphere assay, the effect of soluble laminin was investigated. After 21 days in culture, two-fold more primary neurospheres had formed in the presence of laminin compared to medium alone (Fig. 2A; *p *< 0.05, n = 2). When these primary neurospheres were dissociated and replated at the same clonal density but now in the absence of added laminin, no difference in the number of secondary neurospheres produced was observed (Fig. 2B; 8.63 ± 1.00 versus 7.00 ± 0.82 neurospheres per 500 cells for cells *originally *grown in medium or medium+laminin, respectively). Therefore, laminin increased primary neurosphere formation, but had no effect on secondary sphere growth. This shows that the relative numbers of hNSC was not increased by growth in laminin, suggesting that the effect of the laminin responsible for increased numbers of primary spheres is on NSC proliferation/survival.

### Augmented cell survival in the presence of laminin

The loss of attachment to the ECM usually leads to cells undergoing anoikis [28]. Consequently, laminin in the culture medium may prevent cells from undergoing apoptosis, leading to increased primary neurosphere formation. In order to investigate this, human neurosphere cells were treated with laminin for 24 hrs before being stained for the presence of phosphatidylserine in the external leaflet of the plasma membrane, a marker of apoptosis, by FITC-conjugated annexin V (Fig. 2C) [29]. The resulting data were normalised to the values seen with medium alone. Laminin led to a decrease in annexin V staining, with 73.69 ± 4.70% of cells being annexin V^+ ^relative to medium alone (*p *< 0.01; *n *= 5). As such, laminin decreased apoptosis in cells dissociated from human neurospheres.

### Cell survival is mediated by integrin β1

Integrins are the main receptor family for the ECM, with α3β1, α6β1, α7β1 and α6β4 mostly responsible for binding laminin [10]. The role of integrin subunit β1 in mediating the effect of laminin on cell survival was investigated further for two reasons (Fig. 2C). Firstly, integrin subunit β4 is not expressed by human neurospheres [30]. Secondly, in parallel experiments using the much faster-growing murine cells we found that integrin β1-blocking antibodies negated the laminin-induced increase in neurosphere numbers (see Additional file 3 and Additional file 1). In the absence of laminin, adding an isotype control or integrin β1-blocking antibody to the human cell cultures did not significantly alter the percentage of annexin V^+ ^cells relative to medium alone (99.52 ± 9.44% and 115.90 ± 11.81%, respectively). However, in the presence of laminin, the integrin β1-blocking antibody led to a significant rise in the percentage of annexin V^+ ^cells, increasing from 80.74 ± 2.03% in the presence of the isotype control antibody to 96.18 ± 0.94%, relative to medium alone (*p *< 0.05, *n *= 3). Therefore, integrin β1 is necessary for the laminin-mediated increase in survival.

### Integrin activation is not sufficient to recapitulate the laminin effect

**Figure 3:**
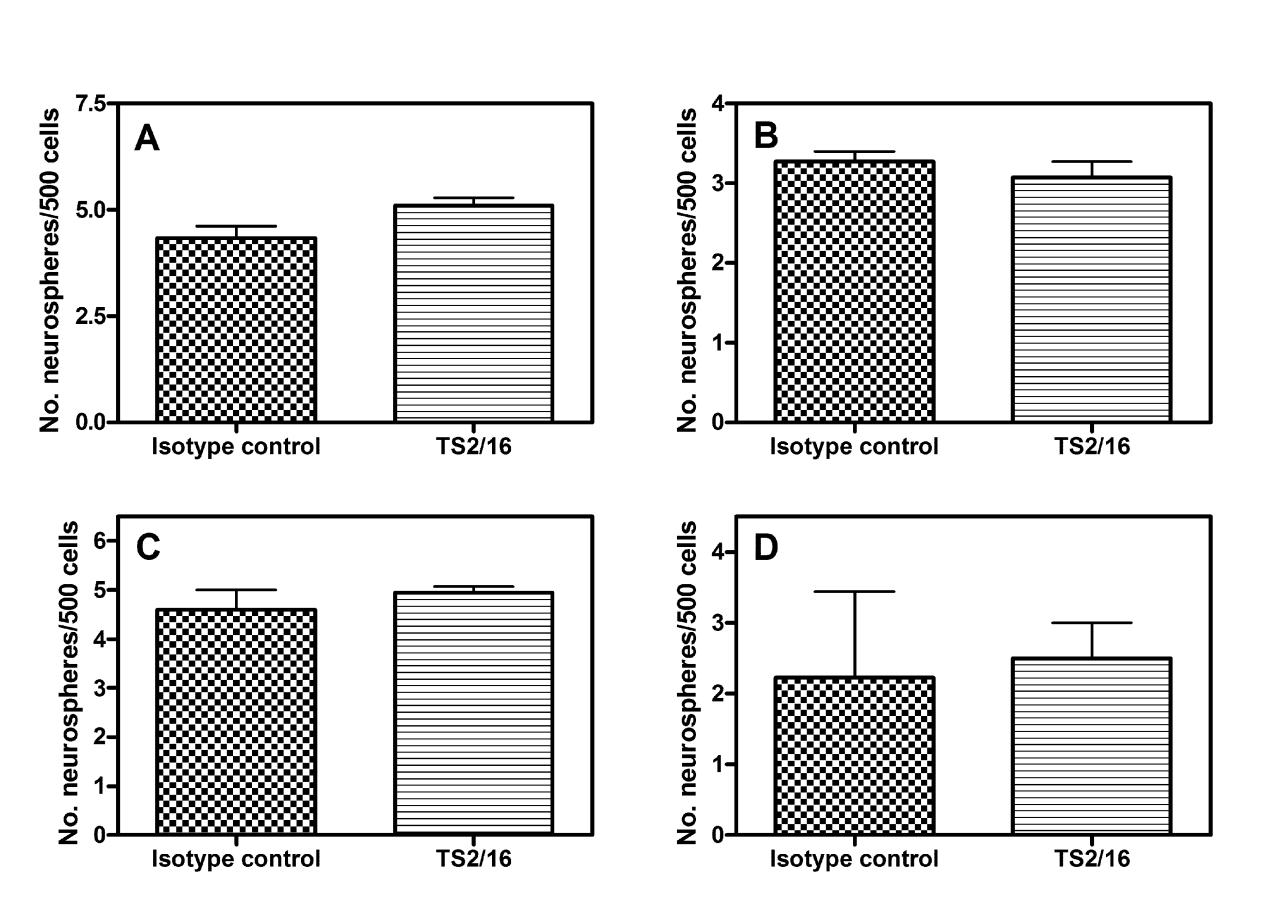
**Activation of integrin β1 does not recapitulate the effects of laminin**. (A) Cells were dissociated from human neurospheres and plated at 500 cells per well in the presence of medium supplemented with either an isotype control antibody or a β1 integrin stimulatory antibody (TS2/16; both at 10 μg/ml). Note that no alteration to the number of primary neurospheres which formed was observed. (B) Primary neurospheres were dissociated and re-plated in medium alone. Again, TS2/16 had no effect on secondary neurosphere formation. (C) As for (A), except that the cells were grown in 2 ng/ml EGF and FGF-2, rather than 20 ng/ml. (D) Secondary neurosphere formation from the primary neurospheres in (C). Statistics were determined by a one-way ANOVA with Tukey post-test. (A) *n *= 5; (B) *n *= 4; (C, D) *n *= 2.

Human laminins are commonly isolated from placenta [31]. This presents three problems to its use by the biotechnology sector. First is the question of how to guarantee sufficient supply. Secondly, laminin placental preparations are heterogeneous. Depending on the purification protocol, they contain different laminins that are degraded to varying degrees, and in addition may contain fibronectin [32]. Thirdly, there may be significant variation between batches from the same manufacturer [32]. In contrast, the mouse Engelbreth-Holm-Swarm (EHS) tumour produces relatively pure and un-degraded type of laminin (laminin-111) [33]. However, in light of the expression of non-human antigens by human embryonic stem cells exposed to animal-derived culture supplements [34], it is not suitable for growing cells to be used in therapies. An alternative, more homogeneous and scaleable solution is to use a monoclonal antibody to stimulate the laminin receptor. Given the finding that laminin-dependent survival was mediated by integrin β1, the question of sufficiency was addressed using the integrin β1-activating antibody TS2/16 [35]. The concentration of TS2/16 used (10 μg/ml) was sufficient to activate integrin signalling, as demonstrated by augmented integrin β1-dependent platelet adhesion and the increased phosphorylation of tyrosine 397 on focal adhesion kinase in human neurosphere cells (see Additional file 4 and Additional file 1) [36,37]. However, when added to the hNSC culture medium, no significant difference was observed in the number of primary (Fig. 3A) or secondary (Fig. 3B) neurospheres that formed, compared to an isotype control antibody. Therefore, β1 integrin activation alone was not sufficient to increase neurosphere formation.

Growth factor and integrin signalling demonstrate considerable cross-talk [36]. For example, high levels of platelet-derived growth factor (PDGF) negate any differential effects of ECM substrates on oligodendrocyte proliferation [38]. As all cultures thus far had been supplemented with 20 ng/ml EGF and FGF-2, we examined whether a lower concentration (2 ng/ml) would allow any effects on neurosphere formation of activating integrin signalling to be observed (Fig. 3C, D). However, no significant effects of the activating antibody were found at these lower growth factor concentrations, either for the number of primary (Fig. 3C) or secondary neurospheres produced (Fig. 3D).

## Discussion

The aim of this study was to investigate the role of laminins in promoting human NSC growth. Laminin was found to increase neurosphere formation and cell survival in an integrin β1-dependent manner. However, integrin β1 signalling alone was insufficient to recapitulate the effect of laminin.

### Culture medium requirements for growth of single hNSC

The increase in human neurospheres in the presence of conditioned medium supports the observation that astrocyte-conditioned medium augments the proliferation of mouse NSC [39-41]. The presence of astrocytes within human neurospheres suggests that they could play a similar role [42]. Whilst the identity of the secreted factors in human neurosphere-conditioned medium remains to be elucidated, candidates identified from rodent studies include cystatin C, galectin-1 and pigment-derived epithelial factor (PEDF) [43,44].

We show here that the addition of B27 supplement also enables hNSC to generate neurospheres when plated at low, clonal densities. B27 supplement is chemically defined, containing the all ingredients present in N2 supplement, as well as additional factors. However, it is difficult to assess whether the effect of B27 is due to an individual factor, or the synergy of several. Interestingly, B27 also contains the antioxidant enzymes superoxide dismutase and catalase, which reduce oxidative stress and promote cell survival [45,46]. Although human NSC have been grown at low densities in other studies without B27 supplementation, this has been achieved by using the additional mitogen, leukaemia inhibitory factor (LIF), as well as EGF and FGF-2 [47]. LIF has been demonstrated to have a powerful positive effect on hNSC proliferation via the JAK-STAT pathway and it would be interesting to see if compounds in B27 acted through similar mechanisms [48].

### Laminin promotes hNSC survival

Stem cell maintenance involves two issues: firstly, a proliferative/survival component, and, secondly, the preservation of the undifferentiated state [49]. In this study, laminin led to an increase in primary neurosphere numbers, but had no effect on secondary sphere formation. This suggests that the effect of laminin was on NSC proliferation or survival, rather than promoting the undifferentiated (stem cell) state, as the latter would be predicted to increase the number of secondary neurospheres formed form dissociated primary spheres. In agreement, laminin was found to decrease apoptosis in neurosphere cells in an integrin β1-dependent manner. These findings support work done by Leone and colleagues, who used Cre-LoxP technology to excise integrin β1 from neural cells *in vitro *[50]. They found that stem cell maintenance was unaffected by the loss of the integrin, whilst cell death was increased and proliferation was reduced. Preliminary proliferation data indicates that laminin does not alter the proportion of cells in S or G_2_/M phase (as determined by propidium iodide incorporation and flow cytometry), whereas the addition of EGF/FGF-2 does increase this proportion. Therefore, whilst laminin-integrin β1 interactions mediate hNSC survival, a different ECM-integrin combination appears to regulate proliferation.

At least two waves of apoptosis occur during CNS development. Cell death is prominent amongst proliferating neural stem/precursor cells and neuroblasts present in the ventricular and intermediate zones in both rodents and humans [51]. At later developmental stages, cell death mainly happens in post-mitotic neurons that have failed to innervate their targets [52]. The importance of cell death in the stem/precursor populations is shown by knock-outs of various components of the apoptotic pathway in mice. These result in the malformation of the CNS, with enlarged ventricular zones and disruption of the cytoarchitecture [53-55]. As these alterations are apparent from E9.5–E10.5, before neurogenesis commences, they must reflect a requirement for regulated cell death in the neural stem/precursor cells as part of normal development.

The function of cell death at this early stage of CNS development is uncertain, although the correction of spatial/temporal errors, control of cell numbers or the selection for particular neuronal phenotypes have all been suggested (reviewed in [56,57]). The control of cell numbers is particularly important, as mutations that lead to the early loss of NSC during development cause microcephaly [58]. Likewise, the upstream pathways are not widely understood, although there are links with known regulators of CNS development. For example, the receptor Patched signals for cell death in neuroepithelial cells unless it is bound by its ligand, Sonic hedgehog (Shh) [59]. Similarly, signalling from receptor tyrosine kinases, via mitogen-activated protein kinase (MAPK) and Akt family members, also regulate apoptosis in the developing neuroepithelium [60-64]. This provides a potential link between the extracellular matrix and apoptosis, as integrins can activate MAPK pathways in rodent NSC [8].

### Laminin-mediated effects require signals additional to integrin β1 activation

Integrin β1 activation failed to reproduce the increased human NSC growth seen with laminin. There are three potential explanations. Firstly, integrin β1 can form heterodimers with subunits α1–11, and αV, which together can bind many ECM molecules including laminins, collagens, tenascins and fibronectin [10]. The different ECM molecules have a different effect on NSC behaviour [23,65]. Therefore, it is possible that activating different integrin β1 heterodimers results in opposite effects, leading to no net increase in NSC growth.

Secondly, integrin signalling is determined by both affinity regulation, through conformational changes in the subunits, and by avidity regulation, in which integrins are clustered. Laminin may form a polymer, presenting multiple integrin binding sites and thus leading to both affinity and avidity changes [28]. In contrast, it is likely that the activating β1 integrin antibody increased affinity only and did not cause integrin clustering. As it has been shown that both processes are required for effective outside-in signal transduction [66], the activating antibody may not result in as complete a signalling response as seen with laminin even though the blocking antibody shows the integrin to be necessary for this signalling.

Thirdly, laminins have been shown to bind to a range of receptors, including dystroglycan and syndecans [28,67]. Dystroglycan is expressed in both the VZ and cortical plate of the embryonic brain [9]. However, mutant mice have defects in neuronal migration, consistent with the cobblestone (type II) lissencephaly phenotype seen in human congenital muscular dystrophy [68,69]. Therefore, it is unlikely that dystroglycan is involved in NSC regulation. However syndecans are also expressed in the VZ and co-operate with integrins in the adhesion of embryonic fibroblasts to fibronectin [9,70,71]. It remains to be examined whether such an interaction occurs in the response to laminin by neurosphere cells and whether syndecans and integrins together promote signalling in NSC in response to ECM.

### Limitations of the neurosphere assay

The neurosphere assay we have used here represents a rapid and convenient method for assessing the number of neural stem cells within a population. However, it is not without its caveats. The main limitation is that cell types other than neural stem cells may proliferate and form spheres, based on the finding that hippocampal progenitors are able to form neurospheres which can be maintained for up to three passages [72,73]. Similarly, transit-amplifying type C cells in the adult brain can also produce neurospheres [74,75]. Consequently, counting the number of neurospheres is not necessarily the same as counting the number of stem cells present in a population. Therefore, the absolute numbers reported in this study may be an over-calculation, with an effect of laminin on the progenitors also possibly contributing to the increased numbers of spheres seen. In order to distinguish the growth of progenitors from NSC, maintaining neurosphere cultures for at least five passages has been suggested, although the exact number of passages required remains debatable [76]. The best way to prove that any effect is directly on the stem cells would be an *in vivo *method such as the repopulating assay used for haematopoietic stem cells, but this has yet to be adapted for the nervous system.

The assay we have used also does not address the impact on multipotentiality of growing hNSC in laminin. The priming of hNSC with FGF-2/heparin/laminin prior to *in vitro *or *in vivo *differentiation resulted mainly in neurons forming, whereas the fate of unprimed hNSC was biased towards an astrocytic phenotype [77]. This is consistent with the findings of other groups that laminin induces a neuronal fate [78]. Moreover, laminin has also been implicated in altering the subtype of neuron formed. Human NSC primed as described above mostly formed cholinergic neurons, in addition to smaller numbers of glutamatergic and gamma-aminobutyric acid (GABAergic) neurons [77]. Similarly, laminin has been found to increase the ratio of GABAergic to glutamatergic neurons compared to fibronectin [79]. Therefore, it would be expected that the culture of hNSC in laminin would bias the cells towards a neuronal fate.

## Conclusion

The aim of this study was to examine the role of laminin signalling in promoting human NSC growth, using an *in vitro *assay to isolate the cells from other niche-derived factors. Laminin was shown to increase primary neurosphere formation and the survival of human NSC. Integrin β1 was necessary for this process, but activation of this integrin was not sufficient. Therefore, multiple receptors or receptor complexes may be necessary in order for laminin to promote hNSC growth. As it is likely that the human placental preparation used in this study contains a number of laminins including laminin-511 (LN-10, α5β1γ1) [32], further work using recombinant laminins is required to define the active laminin isoform and generate reagents suitable for expanding hNSC appropriate for therapeutic use.

## Abbreviations

CNS: Central nervous system; ECM: Extracellular matrix; EGF: Epidermal growth factor; FGF-2: Fibroblast growth factor-2; FITC: Fluoroscein isothiocyanate; (h)NSC: (human) Neural stem cells; LDA: Limiting dilution assay; LIF: Leukaemia inhibitory factor; SEZ: Subependymal zone; SVZ: Subventricular zone; VZ: Ventricular zone.

## Authors' contributions

PEH assisted in study design, conducted the experiments, participated in data analysis and co-wrote the manuscript. JDL assisted in study design, conducted the mouse neurosphere experiments with PEH, participated in data analysis and edited the manuscript. MAC and CffC conceived and designed the study, directed PEH in his practical work and co-wrote the manuscript. All authors read and approved the final manuscript.

## Supplementary Material

Additional file 1 Additional methods. Click here for file

Additional file 2 **Comparison of the effect different laminin concentrations on mouse neurosphere numbers**. Mouse neurospheres were dissociated and plated at 500 cells/well in medium containing 5–20 μg/ml laminin. After 7 days, the number of neurospheres formed was quantified. Compared to wells with no added laminin, significant increases were observed for all concentrations (p < 0.01). However, whilst 10 μg/ml laminin produced significantly more neurospheres than 5 μg/ml (p < 0.01), no significant difference was found between 10 or 20 μg/ml laminin. Therefore, 10 μg/ml laminin was used for all experiments on human cells. Statistics were determined by a one-way ANOVA with Tukey post-test, with 8 technical replicates performed within a single experiment. Click here for file

Additional file 3 **Laminin increases mouse neurosphere formation in an α6β1-dependent manner**. (A) Mouse neurospheres were dissociated and plated in medium containing 10 μg/ml laminin ('Medium+Laminin'), resulting in augmented primary neurosphere formation after 7 days in culture. (B) However, this difference was not maintained upon passaging in medium alone (secondary neurosphere formation). (C) Graph demonstrating that the effect of laminin on primary neurosphere formation is mediated by integrin β1. In medium alone, neither an isotype control antibody ('Iso. cont.') nor an integrin β1-blocking antibody ('β1 block'; 1 μg/ml) affected neurosphere formation. Significantly, though, in the presence of laminin the β1 integrin-blocking antibody reduced neurosphere formation to control levels. (D) Similarly, an α6 blocking antibody ('α6 block'; 20 μg/ml) inhibited the effect of laminin ('Laminin') on neurosphere formation, although blocking another laminin-binding integrin, α7 ('α7 block'; 10 μg/ml) did not. ITC, isotype control (A) *p < 0.0001, as determined by Student's *t*-test; *n *= 3. (C, D) *p < 0.001, as determined by a one-way ANOVA with Tukey post-test; *n *= 3. Click here for file

Additional file 4 **TS2/16 does activate integrin beta 1 signalling**. (A) Platelet adhesion assay, demonstrating that the activating-β1 integrin antibody, TS2/16, stimulates adhesion to pepsin-digested collagen I at the concentrations of 5 μg/ml, relative to medium alone or to an isotype control antibody. (B) Western blot showing that 10 μg/ml TS2/16 stimulates focal adhesion kinase (FAK) phosphorylation on tyrosine 397 (pY397) after 10 min of treatment. 'Iso. Cont.', isotype control antibody (10 μg/ml). *p < 0.01, relative to the isotype control or medium alone, as determined by a one-way ANOVA with Tukey post-test; *n *= 2. Click here for file
