# Supplementary material for: Laminin enhances the growth of human neural stem cells in defined culture media
Source: BMC Neurosci. 2008 Jul 23;9:71. doi: 10.1186/1471-2202-9-71 (PMC2496909; doi:10.1186/1471-2202-9-71)
Supplement: Additional file 1 — Additional methods. [file 1471-2202-9-71-S1.doc]

**Additional methods.**

*Reagents*

Papain was purchased from Lorne Laboratories (Reading, UK). The following antibodies are used at the concentrations indicated in the figure legends: Hamster anti-integrin β1 (clone Ha2/5; BD Pharmingen, Oxford, UK), rat anti-integrin α6 (clone NKI-GoH3; Millipore), mouse anti-integrin α7 (clone 6A11; MBL International, Woburn, USA), hamster IgM isotype control (BD Pharmingen), mouse IgG_1_ isotype control (clone W3/25; Serotec, Oxford, UK) and rat IgG_2a_ isotype control (Serotec). For the western blotting, rabbit anti-phosphorylated tyrosine 397 focal adhesion kinase was purchased from Santa Cruz Biotechnology (Heidelberg, Germany) and rabbit anti-focal adhesion kinase from Biosource (Nivelles, Belgium). All other reagents were from Sigma-Aldrich (Poole, UK)

*Mouse cell culture*

Neurosphere cultures were prepared from the cerebral cortices of embryonic day 15 (E15) CD1 mice. The tissue was dissociated by papain, together with DNase I and L-cysteine (5:3:1), dissolved in Minimal Essential Medium (MEM) at 37°C for 1 h. The digestion was stopped by adding an equal volume of trypsin inhibitor solution (50 µg/ml bovine serum albumin, 40 µg/ml DNase I, 1 mg/ml trypsin inhibitor in L-15 medium), before the cells were collected by centrifuging for 5 min at 180 x *g*. Cells were resuspended in DMEM/Hams F12 (1:1), supplemented with 2% B27, 20 ng/ml EGF, 20 ng/ml FGF-2 and 5 µg/ml heparin. The culture medium was changed every 3 days.

*Platelet adhesion assay*

To perform platelet adhesion assays, human platelets were prepared from fresh, whole, citrated blood (National Blood Service, Cambridge, UK). Platelet-rich plasma was prepared from fresh whole blood by spinning at 200 x *g* for 12 min. Following the addition of 10% (v/v) ACD buffer (39 mM citric acid, 75 mM tri-sodium citrate·2H_2_O, 135 mM D-glucose, pH 4.5) and prostaglandin E_1_ (100 ng/ml final concentration), the platelet-rich plasma was spun at 1000 x *g* for 8 min. The platelet pellet was resuspended in 5 ml of buffer (5.5 mM D-glucose, 128 mM NaCl, 4.26 mM Na_2_HPO_4_·2H_2_O, 7.46 mM NaH_2_PO_4_·2H_2_O, 4.77 mM tri-sodium citrate·2H_2_O, 2.35 mM citric acid, 0.35% bovine serum albumin (BSA), pH 6.5). Prostaglandin E_1_ was added as before, and the platelets were spun at 700 x *g* for 8 min. Platelets were resuspended to either 1 x 10^8^ or 5 x 10^7^ platelets/ml in either adhesion buffer (0.05 M Tris-HCl, 0.14 M NaCl, 0.1% BSA, pH 7.4) or modified Tyrodes buffer (10 mM HEPES-NaOH, 138 mM NaCl, 5.6 mM glucose, 0.4 mM Na_2_HPO_4_·2H_2_O, 12 mM NaHCO_3_, 2.7 mM KCl, 0.35% BSA). Platelets were left to rest for at least 20 min at room temperature. Prior to use, platelets were sequentially incubated with the following: 2 μM GR144053F for 5 min to reduce platelet-platelet interactions by blocking αIIbβ3 (a kind gift of GlaxoSmithkline, Stevenage, UK), 2 mM MgCl_2_ and CaCl_2_ for 5 min, and antibodies for 15 min.

96-well plates (Immulon 2, Dynex Technologies, Ashford, Middlesex, UK) were coated with 100 µl per well of monomeric type I collagen (20 µg/ml) in 0.01M acetic acid for 1 hr at 20°C. Ligand-coated wells were blocked by incubation with 200 µl of blocking buffer (0.05 M Tris-HCl, 0.14 M NaCl, 5% BSA, pH 7.4) for 30 min. The wells were washed three times with 200 µl of adhesion buffer, and then 50 µl of platelet suspension was added to each well and left for 1 hr. The wells were emptied and washed three times with 200 µl of adhesion buffer to remove non-adherent platelets. Adherent platelets were lysed by incubation for 1 hr with 150 µl per well of lysis buffer (0.07 M tri-sodium citrate, 0.3 M citric acid, 0.1% Triton X-100 (v/v), 5 mM *p*-nitrophenyl phosphate). The reaction was terminated by the addition of 100 µl of 2 M sodium hydroxide to each well. Adhesion was measured colourimetrically as the absorbance of the *p*-nitrophenol product at 405 nm in a Maxline Emax microplate reader (Molecular Devices Ltd., Crawley, UK). Values were corrected for background by subtraction of readings from BSA-coated wells. The relationship between absorbance and platelet numbers is linear up to 3.0 [72].

To examine the phosphorylation of focal adhesion kinase (FAK) in human neurospheres in response to the integrin activating antibody, the spheres were dissociated by incubation with Accutase (Chemicon) for 15 min at 37°C before trituration. The cells were then starved in DMEM alone for 1 hr at 37°C, followed by the addition of antibody (10 µg/ml) for 10 min. Consequently, the cells were lysed at 37°C for 30 min in TEN buffer (50 mM Tris-HCl, 5 mM EDTA, 150 mM NaCl) containing 1% triton-X100. Protein concentration was determined using the DC protein assay (Bio-Rad). Equal amounts of protein were loaded onto 10% polyacrylimide gels (Bio-Rad) and separated by SDS-PAGE before being transferred to a nitrocellulose membrane. Membranes were blocked with 5% bovine serum albumin (BSA) in tris-buffered saline (TBS) with 0.1% tween-20 (blocking buffer) for 1 hr at room temperature. This was followed by the addition of the anti-phosphorylated tyrosine 397 FAK antibody (Santa Cruz Biotechnology, used at1:1000) in blocking buffer overnight at 4°C. After washing, the horseradish-peroxidase conjugated secondary antibody was added for 1 hr at room temperature. The bands were visualised by exposing film to the membranes after treatment with ECL reagent (GE Healthcare). Subsequently, the films were developed using a Xograph Compact X4 x-ray film developer (Tetbury, UK). In order to confirm equivalent levels of FAK in the different lanes, the membrane was stripped using a low pH buffer (0.2 M glycine-HCl, 0.5 M sodium chloride, pH 2.2), before being blocked again as before after which the total FAK antibody (Biosource, Nivelles, Belgium) was also used at 1:1000.
